# Hemostatic powder’s battle against a spurting bleeding ulcer: can it win?

**DOI:** 10.1055/a-2610-3096

**Published:** 2025-06-26

**Authors:** Said Al Alawi, Talat Bessissow, Carlo A. Fallone, Jérémie Jacques, Alan Barkun

**Affiliations:** 1 5620 Division of Gastroenterology and Hepatology, McGill University Health Centre, Montreal, Canada; 2 Gastroenterology and Hepatology, CHU Dupuytren, Limoges, France

A 77-year-old woman with a history of peptic ulcer disease presented with melena and a hemoglobin drop to 70 g/L. After resuscitation with intravenous fluids, transfusion of one unit of packed red blood cells, and intravenous pantoprazole infusion, endoscopy revealed an adherent clot (Forrest class IIb lesion) on the gastric greater curvature ( Fig. 1 ). Removal of the clot exposed a small gastric ulcer with active spurting ( Fig. 2 ). Hemostatic powder (Nexpowder; Medtronic) was applied but failed to stop the bleeding until a single endoscopic hemostatic through-the-scope clip was also applied, achieving hemostasis ( Video 1 ). The patient was discharged uneventfully with a scheduled follow-up endoscopy to ensure healing and biopsies to determine *H. pylori* status. Acute upper gastrointestinal bleeding (UGIB) is a common GI emergency, with a 30-day mortality rate of around 2–3% 1 . Various endoscopic hemostatic techniques have been employed for peptic ulcer bleeding in clinical practice and are well-documented in the literature 2 3 . Despite these interventions, rebleeding occurs in approximately 11–16% of patients with ulcers classified as high-risk (Forrest Ia to IIb), with most cases of rebleeding occurring within the first 72 hours 1 . Topical agents are available in the armamentarium of upper GI bleeding with low to moderate evidence for Tc-325 (Hemospray, Cook) but lacking evidence for other newer endoscopic powders and gels 1 2 3 . Although only a case report, our description shows a failure of a new topical agent Nexpowder as first-line treatment of a spurting gastric ulcer bleed followed by immediate subsequent good response using mechanical therapy with through-the-scope endoscopic clips. Active arterial bleeding or “spurting” has already been described by Sung et al. 4 to be one of the more challenging situations when using Hemospray, although a large randomized trial suggested its use as a single first-line agent in peptic ulcer bleeding reporting a success rate of 88.9% in patients with spurting vessels 5 . Further evidence is urgently needed to better define the performance of newer topical agents and optimal lesion selection when used as first-line therapy, as is the case with an ongoing trial using Nexpowder (Registration No. NCT06188585).

**Fig. 1:**
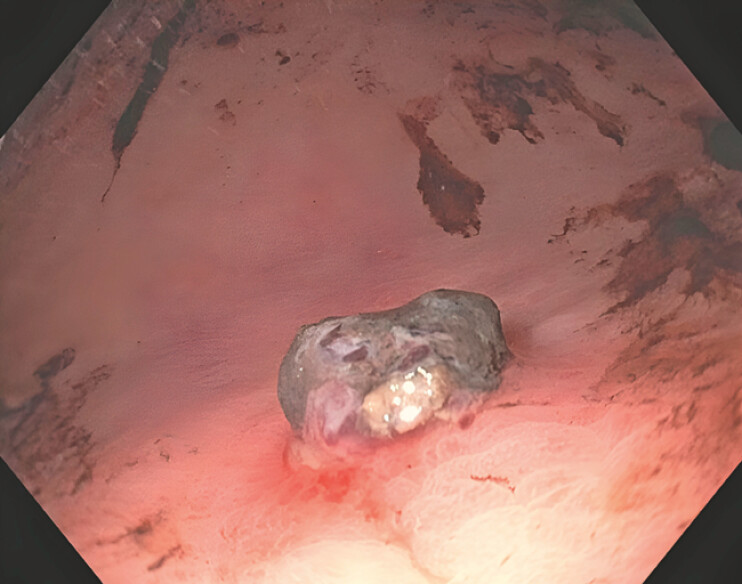
Endoscopy picture of the gastric ulcer with adherent clot, Forrest class IIb.

**Fig. 2:**
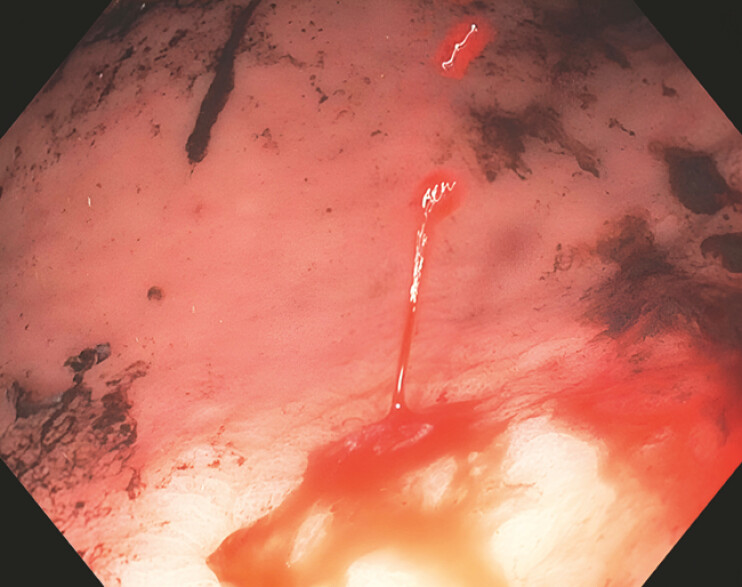
Endoscopic picture of the gastric ulcer with active spurting vessel bleeding, once the clot was removed (Forrest classification IA).

Endoscopic hemostasis for active spurting vessel gastric ulcer.Video 1

Endoscopy_UCTN_Code_CPL_1AH_2AC

## Notes

Endoscopy E-Videos https://eref.thieme.de/e-videos
*E-Videos* is an open access online section of the journal *Endoscopy* , reporting on interesting cases and new techniques in gastroenterological endoscopy. All papers include a high-quality video and are published with a Creative Commons CC-BY license. Endoscopy E-Videos qualify for HINARI discounts and waivers and eligibility is automatically checked during the submission process. We grant 100% waivers to articles whose corresponding authors are based in Group A countries and 50% waivers to those who are based in Group B countries as classified by Research4Life (see: https://www.research4life.org/access/eligibility/ ). This section has its own submission website at https://mc.manuscriptcentral.com/e-videos .
